# New probiotics (*Lactobacillus plantarum* and *Saccharomyces cerevisiae*) supplemented to fermented rice straw-based rations on digestibility and rumen characteristics *in vitro*

**DOI:** 10.5455/javar.2023.j657

**Published:** 2023-03-31

**Authors:** Yetti Marlida, Harnentis Harnentis, Yuliaty Shafan Nur, Laily Rinda Ardani

**Affiliations:** 1 Department of Animal Nutrition, Faculty of Animal Science, Andalas University, Padang, Indonesia; 2 Ph.D. Student (PMDSU Program), Graduate Program Faculty of Animal Science, Andalas University, Padang, Indonesia

**Keywords:** Digestibility, lactic acid bacteria, probiotics, rice straw, rumen characteristics, yeast

## Abstract

**Objective:**

This research was arranged to explore the effect of supplementation of a combination of *Lactobacillus plantarum* and *Saccharomyces cerevisiae* as a new probiotic in fermented rice straw-based rations on *in vitro* digestibility and ruminal characteristics.

**Materials and Methods:**

A randomized group design with 3 types of treatment and 4 replications as a group was used in this study. A probiotic inoculum containing *L. plantarum *and *S. cerevisiae *with 1 × 10^10^ colony-forming unit (CFU)/ml*. *Treatments were followed by: P1 = complete rations without probiotics (control), P2 = P1 supplemented 0.5% probiotics, and P3 = P1 supplemented 1% probiotics. Substrate complete rations were based on the fermented rice straw and concentrate (60%:40%). Parameters of digestibility and rumen fermentation products were determined after 48 h of incubation.

**Results:**

Probiotics supplemented with fermented rice straw-based rations significantly increased (*p* < 0.05) digestibility and rumen characteristics *in vitro*. Supplementation with 1% probiotics (P3) produces the highest digestibility compared to other treatments: in-vitro dry matter digestibility (IVDMD) (55%), in-vitro organic matter digestibility (IVOMD) (58.28%), in-vitro crude protein digestibility (IVCPD) (84.42%), in-vitro acid detergent fiber digestibility (IVADFD) (53.99%), in-vitro neutral detergent fiber digestibility (IVNDFD) (58.39%), and in-vitro cellulose digestibility (IVCLD) (67.12%). Rumen pH (6.76–6.80) did not change significantly (*p* > 0.05) due to supplemented probiotics. Probiotic supplementation in rations significantly (*p* < 0.05) increased the content of NH_3_ and total volatile fatty acid (VFA). Supplementation with 1% probiotic (P3) showed the highest concentration of NH_3_ (26.56 mg/100 ml) and was also followed by the total VFA (115.75 mM) compared to the control (22.59 mg/100 ml and 103.00 mM, respectively).

**Conclusion:**

Supplementation of 1% probiotics (combination of *L. plantarum* and *S. cerevisiae)* containing 1 × 10^10 ^CFU/ml in fermented rice straw-based rations increases nutrient digestibility, that is, IVDMD, IVOMD, IVCPD, IVADFD, IVNDFD, and IVCLD, and also increases rumen fermentation, which is the concentration of NH_3_ and total VFA.

## Introduction

The main problem in animal husbandry is the difficulty of obtaining quality fodder for ruminant breeders such as cows, goats, buffaloes, and sheep. This can reduce the productivity of ruminants. Increasing livestock welfare can be achieved by providing quality feed [1]. The use of agricultural waste can be a strategy to maintain feed availability for ruminants in tropical and subtropical regions [2]. However, the agricultural waste produced in agricultural activities has not been utilized optimally and is a problem in the environment [1]. Utilization of agricultural waste as ruminant animal feed can stimulate the integration of livestock agribusiness, which is commonly called a “zero waste production system” [3].

 In tropical countries with rice-based agricultural systems, rice straw is a potential agricultural waste that can be found in almost all regions. However, many researchers and scientists from various countries are developing alternatives to convert rice straw into a commodity that has more use value and is beneficial to society [4]. Rice straw is an important feed ingredient during the dry season due to the limited forage and other animal feed [5]. However, rice straw has poor palatability and digestibility, high bulkiness, low protein and minerals, high lignocellulose, and insoluble ash, which disturb its utilization as a source of ruminant feed [6]. Dry matter (DM) digestibility is relatively low, as experienced by livestock fed dry rice straw. Biological treatment by adding microorganisms such as probiotics can be chosen because it is economical and increases digestibility [7].

Supplementation of live microorganisms in probiotics can optimize the host digestive tract [8] and increase feed digestibility [9]. Probiotics were chosen because they can reduce the potential risk of residues, reduce the transfer of pathogens to humans, and increase productivity [10]. Probiotic supplementation can help manipulate rumen fermentation and improve the metabolism of carbohydrates, proteins, and fats [10,11]. Among the commonly used probiotics are *Lactobacillus plantarum* (lactic acid bacteria; LAB) and *Saccharomyces cerevisiae* (yeast). LAB as probiotics can increase feed efficiency, interact with rumen microorganisms, increase propionate production and total volatile fatty acid (VFA) [12], and have the potential to act as growth promoters [10]. Supplementation of *S. cerevisiae* can increase the use of cellulose (CLD), consume oxygen, and prevent the overproduction of lactic acid [13]. In addition, *S. cerevisiae* can stimulate rumen cellulolytic and lactate-utilizing bacteria by producing metabolites such as minerals, organic acids, or vitamins [13]. Probiotic microorganisms are selected to reduce and modify lignin structure, increase digestibility, and increase hydrolytic enzymes having the ability to produce sufficient amounts to degrade CLD and hemicellulose structures on the substrate [14]. Several studies have reported that the use of probiotics in rice straw can increase the digestibility of DM and neutral detergent fiber (NDF) and improve the *in vitro* rumen fermentation characteristics of rice straw silage using LAB [15]. Meanwhile, in the report by Selim et al. [16], the addition of mixed probiotics consisting of *L. plantarum, L. bulgaricus, L. acidophilus*,* L. rhamnosus, Bifidobacterium bifidum, Streptococcus thermophilus,* and *Enterococcus faecium* containing 1.8 × 10^8^ colony-forming unit (CFU)/gm can increase the nutritional content of rice straw, maintain normal blood parameters and microbial gut flora.

A probiotic combination consisting of *L. plantarum* and *S. cerevisiae* supplemented into rations to improve feed digestibility, and animal performance has not been widely explored. In some parts of the world, especially in the tropics, information regarding the application of rice straw to increase digestibility and livestock production is still limited. Therefore, this study proposes to explore the effects of the supplementation of probiotics of *L. plantarum* and *S. cerevisiae* at different doses of fermented rice straw-based rations on nutrient digestibility and rumen characteristics *in vitro*.

## Materials and Methods

### Ethical approval

Ethical approval was not used in this study because live animals were not used. 

### Study area and period

This study was held at the Feed Industry Technology Laboratory and Ruminant Nutrition Laboratory in the Faculty of Animal Science, Andalas University, Padang, Indonesia, from November 2020 to February 2021. 

### Inoculums preparation

*Lactobacillus plantarum* and *S. cerevisiae* were collected in the Feed Industry Technology Laboratory, Faculty of Animal Science, Andalas University. *Saccharomyces *cerevisiae was maintained in 10 ml of medium containing liquid Yeast Peptone Dextrose with the following ingredients: 2 gm peptone (Merck KGaA, Darmstadt, Germany), 2 gm glucose (Merck KGaA, Darmstadt, Germany), and 1 gm yeast extract (HiMedia Laboratories Pvt. Ltd., India). The liquid media that had been planted were incubated at temperatures of 35°C–37°C for 24–48 h. Inoculums of *L. plantarum* were grown in 10 ml medium liquid of DeMan Rogosa Sharpe Broth (Merck KGaA, 64271 Darmstadt, Germany), incubated for 24–48 h, and maintained at 37°C. 

### Growing inoculums on natural medium

The combination inoculums of *L. plantarum* and *S. cerevisiae* were then grown on natural media, that is, coconut water, shrimp waste flour, and cassava waste, with a ratio of 90%:5%:5%. Previously, 90 ml of coconut water was put into the Erlenmeyer, and then 5 gm of shrimp waste flour and 5 gm of cassava waste were added. Then it was put into the autoclave for 15 min, and after the cold medium was added, as much as 5% mixed probiotics were allowed to grow on the natural medium and incubated at 37°C for 24 h. The number of colonies of each microbe (CFU) was counted according to the growth medium to ensure that the microbes grew after 24 h of incubation. 

### Experimental design

This study followed the Tilley and Terry method [17] to conduct rumen *in vitro* incubation. In this study, the rumen fluid was taken from a slaughterhouse in West Sumatra, Indonesia. This study used three treatments and four replications with a completely randomized design. The complete ration comprised 60% fermented rice straw and 40% concentrate (tofu waste, bran, palm kernel meal, and minerals) (Table 1). Rice straw is fermented using StarBio and urea for 21 days. Probiotics inoculum was used containing *L. plantarum* and *S. cerevisiae* with 1 × 10^10^ CFU/ml. The treatment was as follows: P1 = complete rations without probiotics (control), P2 = P1 supplemented 0.5% probiotics; and P2 = P1 supplemented 1% probiotics. 

### In vitro method

2.5 gm of complete rations (Table 1) were put into an Erlenmeyer bottle with a capacity of 300 ml and filled with McDougall‘s buffer solution (200 ml) and rumen fluid (50 ml). McDougall‘s buffer solution consisted of NaHCO_3_ 9.8 gm (Merck KGaA, Darmstadt, Germany), Na_2_HPO_4_.7H_2_O 3.68 gm (Merck KGaA, Darmstadt, Germany), KCl 0.57 gm (Merck KGaA, Darmstadt, Germany), MgSO_4_.7H_2_O 0.12 gm (Merck KGaA, Darmstadt, Germany), and NaCl 0.47 gm (Merck KGaA, Darmstadt, Germany). All ingredients are dissolved, which equates to 1 l. The Erlenmeyer bottle was filled with CO_2_ gas to create anaerobic conditions for about ±30 sec. The incubation process lasted 48 h. Then, after incubation, the samples were centrifuged to separate the supernatant and residue for 30 min, 1,509 × gm. Fraction of liquid results was used for NH_3_ and VFA total analysis. Before being used for analysis, samples were stored in a refrigerator at a temperature of −20°C. NH_3_ level was determined by following the Conway and O’Malley method [18], and the total VFA level was determined through steam distillation [19]. The residue from the centrifuge was then filtered with paper (Whatman TM 41 CAT No 1441-125) with a funnel and placed a waste bottle underneath to dispose of the filter results. The residue was dried in the oven for 8 h at a temperature of 60°C. The residue in the oven is a sample that will be used for the analysis. The nutrient content of dried residue was determined following proximate [20] and Goering and Van Soest [21] analysis. *In vitro* digestibility was calculated using these formulas:


$$\text{ IVDMD }=\frac{\text{ DM samples (DM residue }-\text{ DM blanks) }}{\text{ DM sample }}\times 100\%$$

$$\text{ IVOMD }=\frac{\text{ OM samples }-\text{ (DM residue }-\text{ OM blanks })}{\text{ OM sample }}\times 100\%$$

$$\text{ IVCPD }=\frac{CP\text{ samples }-(CP\text{ residue }-CP\text{ blanks })}{\text{ CP sample }}\times 100\%$$

$$\text{ IVADFD }=\frac{\text{ ADF samples }-(\text{ ADF residue }-\text{ ADF blanks })}{\text{ ADF sample }}\times 100\%$$

$$\text{ IVNDFD }=\frac{\text{ NDF samples }-\text{ (NDF residue }-\text{ NDF blanks })}{\text{ NDF sample }}\times 100\%$$

$$\text{ IVCLD }=\frac{\text{ CLD samples }-(DM\text{ residue }-\text{ DM blanks })}{\text{ DM sample }}\times 100\%$$

where:

DM, organic matter (OM), crude protein (CP), acid detergent fiber (ADF), NDF, CLD, *in-vitro* dry matter digestibility (IVDMD), *in-vitro* organic matter digestibility (IVOMD), *in-vitro* crude protein digestibility (IVCPD), *in-vitro* acid detergent fiber digestibility (IVADFD), *in-vitro* neutral detergent fiber digestibility (IVNDFD), and *in-vitro* cellulose digestibility (IVCLD). 

### Statistical analysis

This study used a randomized group design consisting of three treatments with four replications. Observational data were analyzed using Analysis of variance. The results were significantly different, followed by Duncan’s tests.

## Results and Discussion

The result of this study with probiotic supplementation (L. plantarum and S. cerevisiae) as a new probiotic candidate can be observed through nutrient digestibility (Table 2) and rumen fermentation products in vitro (Table 3). Supplementation of probiotics (a combination of L. plantarum and S. cerevisiae) resulted in various digestibility and rumen characteristics of fermentation.

## In vitro nutrient digestibility

Probiotic supplementation (combination of *L. plantarum* and *S. cerevisiae*) on fermented rice straw-based feed had a significantly different (*p* < 0.05) nutrient content digestibility *in vitro*, including DM, OM, CP, ADF, NDF, and CLD (Table 2). The highest *in vitro* nutrient digestibility occurred in the P3 (supplementation with 1% probiotics), which showed results of IVDMD (55%), IVOMD (58.28%), IVCPD (84.42%), IVADFD (53.99%), IVNDFD (58.39%), and IVCLD (67.12%). Meanwhile, for the P2, with 0.5% probiotic supplementation, the digestibility of IVDMD (51.8%), IVOMD (54.8%), IVCPD (77.57%), IVADFD (51.19%), IVNDFD (55.35%), and IVCLD (63.47%) did not show significantly different results from the control.

Probiotic supplementation (combination of *L. plantarum *and* S. cerevisiae*) based on fermented rice straw rations and a concentrate ratio of 60%:40% on nutrient digestibility (IVDMD, VOMD, IVCPD, IVADFD, IVNDFD, IVCLD) is shown in Table 2. Rice straw is poor quality feed in terms of mineral and protein content. In addition, the high content of lignocellulosic and insoluble ash [6]. DM digestibility has correlated positively with OM digestibility because OM is the main component of DM. [22]. ADF, NDF, silica, and lignin are the dominant components of the cell walls of rice straw, which are limiting factors in nutrient degradation, including DM, OM, and CP [23]. CP available in complete rations can be a source of protein for ruminants through the synthesis of microbial protein from protein that can be degraded in the rumen or derived from rumen bypass/undegradable protein [24]. Rumen microorganisms can produce cellulase and hemicellulase enzymes to degrade the NDF fraction. According to the study by Khanday et al. [6], the administration of LAB can decrease the concentration of NDF and ADF, as well as increase the digestibility of DM and NDF from rice straw silage. Lesmana et al. [25] added that ADF components below 30% can still produce good fiber digestibility. Probiotic supplements can help manipulate the rumen environment so that fermenting food substances can be optimal [6]. In addition, probiotic supplementation can reduce ADF content, thereby increasing digestibility and digestible energy [7]. Probiotic supplementation with LAB can digest CLD, hemicellulose, and lignin components during the fermentation process to reduce NDF levels [26] The decrease in CLD and hemicellulose content is thought to be due to probiotic microorganisms being able to break down lignified bonds and fibrinolytic structures to a certain extent [6].

**Table 1.** Ingredients and nutrient composition of the rations.

Item	P1	P2	P3
Ingredients (%)			
Fermented rice straw	60	60	60
Bran	12	12	12
Palm kernel meal	12	12	12
Tofu waste	15	15	15
Mineral premix^a^	1	1	1
Probiotics	-	0.5	1
Nutrient ingredients	Contents (100%DM)		
OM	87.12		
CP	12.30		
Crude fibre	24.18		
Crude fat	4.42		
Ash	12.88		
Total digestible nutrient	64.46		
NDF	53.89		
ADF	33.99		
CLD	24.42		
Hemicellulose	19.90		
Lignin	5.37		
Silica	4.20		

a Mineral premix composition (per kilogram): calcium carbonate 500 gm, phosphate flour 150 gm, manganese sulfate 1.25 gm, potassium iodide 250 gm, cuprum sulfate 0.7 gm, sodium chloride 50 gm, ferrum sulfate 2 gm, zinc oxide 1 gm, magnesium sulfate 60 gm.

**Table 2.** *In vitro *nutrient digestibility of supplemented probiotics (%).

Nutrient ingredients	Treatments			
	P1	P2	P3	SEM
IVDMD	50.76^a^	51.80^a^	55.00^b^	0.83
IVOMD	54.02^a^	54.80^a^	58.28^b^	0.96
IVCPD	74.54^a^	77.57^a^	84.42^b^	1.92
IVADFD	50.42^a^	51.19^a^	53.99^b^	0.64
IVNDFD	53.39^a^	55.35^a^	58.39^b^	0.89
IVCLD	62.24^a^	63.47^a^	67.12^b^	0.83

IVDMD = *in vitro *dry matter digestibility, IVOMD = *in vitro *organic matter digestibility, IVCPD = *in vitro *crude protein digestibility, IVADFD = *in vitro *acid detergent fibre digestibility, IVNDFD = *in vitro *neutral detergent fibre digestibility, IVCLD = *in vitro *cellulose digestibility, P1 = complete rations without probiotics (control), P2 = P1 supplemented 0.5% probiotics, P3 = P1 supplemented 1% probiotics. Superscript^a,b^ significantly different in a row (*p* < 0.05).

This study is in accordance with the previous research, supplementation of probiotic mix containing yeast in paddy straw-based rations increased the digestibility of IVDMD (42.47%), IVOMD (47.08%), and IVNDFD (33.75%) [10]. Another report by Shelim et al. [7] reported that supplementation of Protexin (commercial probiotic) significantly increases the digestibility of CP (62.90% *vs.* 59.20%). Still, in that report, the digestibility of ADF, NDF, DM, and OM was not significantly affected. Increasing digestibility of DM, NDF, and total VFA also happened with the supplementation of *S. cerevisiae* and herb *(Urtica dioica) *in separate and combined forms [27]. Meanwhile, yeast supplementation in bajra straw-based complete rations can increase the digestibility of DM, OM, and NDF and total gas production [28].

The increase in protein digestibility occurred because the microorganisms given to the treatment group were able to secrete extracellular enzymes to break down the protein content in the rice straw and increase enzyme activity in the rumen [29]. The biological treatment of rice straw resulted in a decrease in ADF concentration and NDF, so it is more easily digested by livestock and has a higher nutritional value. As described in the study of Pan et al. [30], who reported that various forages were inoculated with microbial mixtures containing *Bacillus licheniformis* and *B. subtilis* (3.2 × 10^9^ CFU/gm DFM), increasing the average digestibility of the DM and NDF with various quality levels of protein and NDF. The increased digestibility of the fiber fraction is due to the cellulase enzymes secreted by microbial inoculants. This effect was also reported where supplementation of the probiotic microorganism increases the hydrolytic activity of cellulase by breaking the hydrogen bonds between hemicellulose and CLD, thus increasing the accessibility of cellulase to the polysaccharide cell wall [31]. Improved digestibility is also explained by the ability of probiotic microorganisms to provide vitamins that can enhance cellulolytic activity in the rumen, nutritional factors such as enzymes (endopectatelyase, pectin methylesterase, and exopectatelyase), and short-chain fatty acids [32].

## In vitro ruminal characteristics

Supplementation of probiotics (a combination of *L. plantarum *and* S. cerevisiae*) on fermented rice straw-based rations was not significant (*p* > 0.05) on rumen pH value (Table 3). pH values in this research ranged from 6.76 to 6.80. This exploration is also appropriate with Sheikh et al. [10], whose rumen pH resulting from supplementing a probiotic mix containing yeast on paddy straw-based feed was 6.77. Rumen pH stability was also shown in the study of Cai et al. [33], who reported that supplementation with *S. *cerevisiae, *Clostridium butyricum*, or a combination of both was able to stabilize pH conditions in the rumen. Rumen pH under normal circumstances indicated that probiotic supplementation (*L. plantarum *and* S. cerevisiae*) is capable of optimally supporting rumen microbes’ activity. *Saccharomyces cerevisiae* can increase the anaerobic state in the rumen by using oxygen and maintaining the stability of the rumen pH [34]. Live yeast in rations improves and stabilizes rumen pH by providing nutrients that produce some metabolites for bacterial growth, including lactic acid-utilizing bacteria and cellulolytic bacteria [35,33].

However, probiotic supplementation (a combination of *L. plantarum *and* S. cerevisiae*) significantly (*p* < 0.05) affected NH_3_ production and total VFA (Table 3). In this study, the NH_3_ production ranged from 22.95 to 26.56 mg/100 ml. The highest concentration of NH_3_ was shown by P3, 1% probiotic supplementation (26.56 mg/100 ml), compared to other treatments. This result is higher than the report by Sheikh et al. [10], NH_3_ production (17.61 mg/dl) on paddy straw-based probiotic supplementation. An increase in NH_3_ production also occurred in studies using forage substrates supplemented with probiotic *Clostridium butyricum* in batch culture systems [36]. NH_3_, derived from the catabolism of amino acids (AA) and peptides, contributes N to microbes in synthesizing microbial proteins [37]. Microbes use ammonia (NH_3_) as a nitrogen source to produce AA and peptides for livestock growth. Microbial products can be reabsorbed into the circulation of the host mammalia and used for synthesis processes in the bodies of ruminants [37]. NH_3_, urea, other than AA, peptides, and microbial crude protein play an important role in nitrogen digestion and metabolism in ruminants [24]. The resulting microbial protein can be utilized by the ruminant body as a source of AA. However, carbohydrates are needed as the main energy source in microbial protein synthesis [24], so a balance between energy sources and N content is important for the ruminant. ATP derived from carbohydrate metabolism is used for microbial growth [24]. Increased protein digestibility can impact increasing NH_3_ production levels because digested proteins can fulfill the availability of N sources so that they contribute better to microbial protein synthesis [33].

**Table 3.** *In vitro *ruminal characteristics of supplemented probiotics.

Parameter	Treatments			
	P1	P2	P3	SEM
pH	6.80	6.77	6.76	0.04
NH_3 _concentration (mg/100 ml)	22.95^a^	23.91^a^	26.56^b^	0.57
Total VFA (mM)	103.00^a^	105.75^a^	115.75^b^	2.18

NH_3_ = ammonia, VFA = volatile fatty acid, P1 = complete rations without probiotics (control), P2 = P1 supplemented 0.5% probiotics, P3 = P1 supplemented 1% probiotics. Superscripts ^a,b^ are significantly different in a row (*p *< 0.05).

We observed an increase in total VFA production in the group supplemented with probiotics (a combination of *L. plantarum *and* S. cerevisiae*) based on fermented rice straw rations (Table 3). The highest total VFA was shown by P3, 1% probiotic supplementation (115.75 mM). Treatment without probiotics (the control) showed the lowest results (103.00 mM), followed by P2, 0.5% probiotic supplementation (105.75 mM), which was statistically not significantly different from the control. Probiotic supplementation can manipulate rumen fermentation [38]. The increase in total VFA is due to probiotic supplementation [10,39]. Consistent with the investigation of Jiao et al. [40], increased levels of VFA and DM digestibility occurred due to increased supplementation of live yeast in rations. Supplementation of probiotics can stimulate fiber-degrading microbes so that there is more energy availability [10]. VFA provides the largest energy supply for ruminants, where VFA production indicates a variety of roles for fibrinolytic or amylolytic microorganisms and describes the metabolic status of rumen microbes [38]. Research by Sheikh et al. [10] produced total VFA (79.81 mEq/l) on paddy straw-based probiotic supplementation. Supplementation (*S. cerevisiae* 2 × 10^10 ^CFU/gm) and herb (*U. dioica) *can increase VFA and NH_3_ in rice straw-based feed [27]. The increase in total VFA concentration in this study confirms that probiotic microorganisms can stimulate microbial activity in the rumen, especially fibrinolytic bacteria [33]. A large number of microorganisms in the rumen carry out the fermentation of carbohydrates and non-structural carbohydrates to produce VFA and microbial protein synthesis for host livestock. The fermentation process, which increases rumen digestion, absorption capacity, and metabolism of nutrients, is critical to the nutritional supply of ruminants [41].

Based on the findings, supplementation of 1% probiotics (combination of *L. plantarum* and *S. cerevisiae*) in P3 containing 1 × 10^10 ^CFU/ml consistently showed the highest *in vitro* nutrient digestibility and rumen characteristics. However, further studies are still needed for implementation in terms of reducing methane gas production and microbial diversity resulting from the dose that has been found. In addition, it is also necessary to conduct *in vivo* studies on ruminants to determine the impact of giving probiotics on livestock productivity.

## Conclusion

Supplementation of 1% probiotics (a combination of *L. plantarum* and *S. cerevisiae*) in P3 containing 1 × 10^10^ CFU/ml in fermented rice straw-based ratios consistently showed the highest *in vitro* nutrient digestibility and rumen characteristics. This supplementation showed *in vitro* digestibility of IVDMD (55%), IVOMD (58.28%), IVCPD (84.42%), IVADFD (53.99%), IVNDFD (58.39%), and IVCLD (67.12%). Meanwhile, supplementation of 1% probiotic (P3) also increases the product of rumen characteristics, which is a concentration of NH_3_ (26.56 mg/100 ml) and total VFA (115.75 mM). This exploration shows that probiotic microorganisms can manipulate the rumen environment by encouraging microbial activity, which in turn can degrade feed ingredients so that rumen characteristics and digestibility increase. In future studies, it is important to explore an *in vivo* study to see ruminants’ productivity when fed fermented rice straw-based rations supplemented with this probiotic combination. 

## List of Abbreviations

ADF, Acid detergent fiber; ATP, Adenosine triphosphate; CFU, Colony-forming unit; CLD, Cellulose; CP, Crude protein; DM, Dry matter; IVADFD, *in vitro *acid detergent fiber digestibility; IVCLD, *in vitro *cellulose digestibility; IVCPD, *in vitro *crude protein digestibility; IVDMD, *in vitro *dry matter digestibility; IVNDFD, *in vitro *neutral detergent fiber digestibility; IVOMD, *in vitro *organic matter digestibility; NDF, Neutral detergent fiber; NH_3_, Ammonia; OM, Organic matter; VFA, Volatile fatty acid.
